# Report of Discussions at the Twenty-Fourth Annual Meeting of the Mississippi Valley Dental Association, March 4, 5 and 6, 1868

**Published:** 1869-05

**Authors:** 


### Proceedings of Societies.

---

### REPORT OF DISCUSSIONS AT THE TWENTY-FOURTH ANNUAL MEETING OF THE MISSISSIPPI VALLEY DENTAL ASSOCIATION, MARCH 4, 5 AND 6, 1868.

The first subject presented for consideration was "What are the best methods of controlling flow of saliva during the operation of filling teeth?"

Dr. GODDARD remarked that inability to control the saliva in the mouths of his patients, he had found one of his greatest difficulties in filling teeth. Has found it impossible in many cases to keep the mouth dry by any of the ordinary methods. He had been for a few days using the rubber dam, in connection with the saliva pump, and thinks it has, in some cases, advantages over any other method.

Dr. JAMES TAYLOR finds very great difficulty in keeping the mouth dry while operating; it sometimes seems as though the saliva flows in great quantity through all the ducts, into the mouth, and to prevent flooding is next to impossible. The breath is oftentimes so loaded as to completely moisten the filling when it is permitted to come in contact with it.

Dr. BERRY thinks by heating and keeping the gold slightly warmer than the mouth, during the introduction of the filling, the moisture from the breath will be obviated; employs the ordinary means for the exclusion of the saliva.

Dr. McCLELLAND has discarded the use of saliva pumps thinks they are useless for keeping away saliva; relies upon a good supply of napkins, properly employed, and in connection with them uses "Hawes Tongue Holder;" especially is this arrangement applicable for the inferior molars of the left side, and in addition to this, while operating, inclines the head of the patient to the right; for the inferior molars of the right side, holds the napkins in proper position about the teeth with the fingers, inclining the head to the left side; removes the saliva often from the mouth, by wiping out with the napkin; has found that sensitiveness of the dentine increases the flow of the saliva; hence, endeavors to obtund that before filling, and for this purpose usually employs creosote; success depends very much upon having all things in readiness, and a good assistant is almost invaluable.

Dr. TAYLOR expressed a desire to know more about the rubber dam; has used the tongue holder twenty-five years; much depends upon the ability of the patient to retain the instrument in its proper position, so it will best secure the parts in the desired position, and not impede the work of the operator.

Dr. TAFT: In deciding upon the method of controlling the saliva in the mouth during an operation, there are several conditions and circumstances that must be taken into account, such as the location of the point to be operated upon, the condition of the dentine and the tooth as a whole, the extent of the decay, the amount of saliva, and its character, the point from which it most freely flows, and the ability of the patient to keep the parts quiet.

The variations attending these conditions indicates to us very clearly that no single method will in all cases, or even in many cases, accomplish the desired object. Sometimes a small amount of saliva, owing to its peculiar condition, will be far more difficult to control than a far greater quantity of a different character. A constant movement of the muscles of the mouth and throat add very greatly to the difficulty of excluding saliva from an operation. Has used almost every method and appliance that has ever been suggested or brought to the notice of the profession, and finds something, and usually much, to commend in almost every one of them; uses napkins much in the manner suggested by Dr. McClelland, more than any other single appliance, but very frequently, and usually quite efficiently, employs bibulous and blotting paper, the rubber dam, and two or three forms of saliva pumps, together with the various tongue compressors; but least efficient of any of these, is the old fashioned tongue-holder or speculum, held by the patient, for there is not more than one patient in fifty that will retain them properly in place, but when a little fatigued will relax the hold and then all is lost; regards the rubber dam as a very great acquisition, and one by which some cases that have hitherto proved almost incontrollable, are by it completely manageable. To Dr. Barnum is due the lasting obligation of the profession for the introduction of this material.

Dr. Morgan has used and relied very much upon blotting paper and napkins of fine linen, about eight inches square; folds into the proper shape and packs them in about the teeth, so as to make pressure upon the mouth of the salivary ducts; never permits the instrument to touch the lips.

Dr. Hays described a little appliance in the form of little round pads, made of porous clay and properly biscuited, for closing the mouths of salivary ducts; they are made plano convex and double convex, from one-half to three-fourths of an inch in diameter; others are made crescent shape. The form and size should be governed by the locality they are to occupy; they are the invention of Dr. Southwick, of Buffalo.

Prof. Cutler read an essay on development of the teeth, in which the idea was advanced that the roots of the teeth, and especially the molars, are not fully formed till a period much later than is generally supposed; that at the time the crowns of these teeth seem to be fully developed, the roots have very commonly large cone-shaped openings at their ends, in which, the destruction of the pulps becomes a serious consideration. The removal of pulps from teeth, the roots of which are in this condition, will be liable to occasion very serious injury to the living parts beyond. The careless or inexperienced operator is very liable to pass entirely through the canals. The roots of the teeth are not in many cases completely developed before the age of eighteen or twenty years.

Dr. WATT feels a very great interest in this subject; he would suggest that there is very great variation in different persons in the period of the complete development of the teeth. He has extracted the first permanent molars at the age of six years, and found the roots perfectly formed, and others at ten years imperfect. This difference depends upon variation of the nutritive function and the developing power. Great care should always be exercised in applying arsenious acid in young persons. He referred to a case in which a girl had an incisor broken off, and upon the root wore a pivot tooth nine years, after which the tooth was removed, and the root found incomplete at its end, never having been completely formed, but it has sustained the artificial tooth well during the time, demonstrating the ability and endurance of even these imperfectly formed roots. There would not be as much liability to injury in the use of the mallet for filling such teeth, as by the clumsy, awkward hand pressure that is so frequently employed. He discussed at some length the theory of mallet pressure, as compared with the hand pressure.

Dr. CUTLER: It is almost impossible to fill the canals of roots before their completion, without doing great violence to the living parts beyond. Usually the roots of the teeth are not perfect till the tenth year.

Dr. OLDHAM differs from both Drs. Cutler and Watt; he believes that the canals of imperfectly formed roots, even though they be somewhat conical as described, may be well filled and better with the mallet than by any other method. He claims that greater precision in the introduction and consolidation of gold is obtained by the use of the mallet.

Dr. H. A. SMITH read an essay upon the action of arsenious acid, when applied to the pulps of the teeth.

Dr. MORGAN, in remarks upon the essay, says that arsenious acid will induce sloughing of soft tissues.

Dr. WATT has been accustomed to use arsenious acid for producing sloughing.

Dr. TAFT: This result is not produced without the agent being taken into the tissue thus affected.

Dr. H. A. SMITH suggests that gentlemen may be mistaken in their preconceived opinions.

Dr. J. TAYLOR: How is there sensitiveness if their nerve is dead or the circulation suspended? He prefers the plan of leaving the teeth for two or three weeks after the application of arsenic before filling, that the pulp may slough away and all sensitiveness be destroyed.

Dr. CUTLER has ha l extensive experience in the use of arsenic in medical practice. It will produce extensive sloughing. It is taken into the system and breaks down the red globules of the blood, combining with the iron, thus depriving the blood, so far as this process is accomplished, of one of its necessary elements. The argument to sustain this theory was in part based upon the fact that the hydrated sesqui oxide of iron, combines with and precipitates arsenic with such facility as to constitute the best known antidote, to its poisonous influence.

I have studied the nature and action of arsenious acid with a considerable degree of thoroughness. Do not think that any of the toxicologists have given the correct theory of the action of arsenic. It produces no change in the general structure of the tooth pulp, when applied for its devitalization, but the red blood corpuscles are broken up and destroyed; this is accomplished by the combination of the arsenic with the iron in the blood. The coloring matter of the blood consists in part, at least, of a sesqui-oxide of iron and the arsenic uniting with it forms an arsenuret of iron. It may also have a catalytic influence upon some of the other constituents of the blood. Arsenic is far more liable to be taken up by dentine before the teeth have arrived at mature development, and mischief is far more liable to occur. We know but little of the definite action of poisons.

Dr. McClelland asks if there is not consequent upon the devitalization and decomposition of tooth pulp, a gas formed that acts as an irritant upon the living parts.

Dr. Cutler replied: There will not be gas formed to any appreciable extent, though by the breaking down of the red corpuscles carbonic acid gas may be formed to a slight extent. When the vessels at the point of a root are cut off, the blood that flowed into the pulp will be diverted to some other channel.

Dr. Morgan is very positive that arsenic by osmotic action does pass through the dentine. The enamel is organic structure and possesses vitality, as is shown by the fact that enamel not sustained by living dentine becomes friable and easily broken down.

Dr. Taft remarked that in many cases in which arsenic is used for devitalization of the pulps of the teeth the periosteum becomes more or less affected. This may occur either from the direct influence of the agent upon the tissue, or in part by this and in part by the congestion consequent upon the sudden stoppage of the blood in its natural course through the vessels of the pulp, and its diversion into other channels, or the difficulty may occur entirely from this latter condition. The blood usually, perhaps upon being turned back, finds its way into the veins by anastomosis, but it will sometimes fail in this and then it passes into the cellular tissue through the ruptured or enfeebled walls of the vessels, when irritation ensues.

### SECOND DAY—EVENING SESSION.

(By special request the first hour of the session was occupied by Dr. Watt, in the delivery of a lecture upon nitrous oxide as an anæsthetic, a synopsis of which we endeavor to give in this connection.—Rep.)

Mr. President and Gentlemen: As most of you are more or less familiar with my recent personal history, I make no apology for appearing before this, the oldest Dental Association in the world, without a written communication.

In compliance with request, I propose a converse, for a while, on the preparation and use of protoxyd of nitrogen, or nitrous oxyd, as an anæsthetic. This is a subject of great practical importance to the Dental profession, inasmuch as we are called upon to inflict pain more frequently than general surgeons, and our operations, though fearfully painful, are of such brief duration that it would be almost warrantable to conclude that this anæsthetic was designed for our special use.

Protoxyd of nitrogen, as its name imports, is composed of one equivalent of nitrogen, united with one of oxygen. The proportions, numerically, are about 14 of the former, and 16 of the latter. It will be noticed that these are the chief elements which constitute our atmosphere, the substance under consideration being about twice as rich in oxygen as atmospheric air; and here these elements are chemically combined, while in the atmosphere they are mechanically mixed.

Nitrous oxyd is a gas about fifty per cent heavier than atmospheric air, is colorless, and has a peculiar sweetish taste and odor. Its volume is the same as that of the nitrogen it contains; hence, by loss of oxygen from any cause, it is not reduced in bulk. This is practically worthy of notice. In this gas the elements are held together by a very feeble affinity. Its oxygen is, therefore, very easily separated from it. On this principle it supports combustion almost as readily and well as free oxygen. The oxygen is thus furnished in its nascent state, and is as active as ozone. It is quite probable that it supports respiration on the same principle. There is a popular error among writers that it may be well to notice. It is generally stated about thus:

"Sir Humphrey Davy discovered * * * that it supports respiration for a few minutes. He breathed 9 quarts of it, contained in a silk bag, for 3 minutes, and 12 quarts for rather more than 4; but no quantity could enable him to bear the privation of atmospheric air for a longer period." Now does any one suppose that 12 quarts of *atmospheric air* used in the same way would support respiration more than 4 minutes? If he does, let him try it; and if it fails him, let him be consistent by writing and printing that "no quantity" of atmospheric air will sustain respiration for a longer period. The ox bladder and silk bag experiments of the older chemists amount to little in determining the support to respiration derivable from this gas. They were mainly ascertaining how long a man can breathe his own breath.

It must not be inferred that this protoxyd is a substitute for atmospheric air, far less that it is a better supporter of respiration, as I have often heard claimed by its over-zealous friends. But that it is capable of supporting respiration far beyond what is indicated by the experiments of Davy is now clearly demonstrated by experiment. I have known it to be breathed for an hour, with less than twenty inspirations of atmospheric air during the time. I have many times seen it breathed twenty minutes, without the admission of any air, the quiet state of the patients, their natural complexions, and their after statements proving that they suffered no inconvenience at the time; and, when the gas is pure and properly administered, even for these long periods, the condition of the patient is as unlike asphyxia as can be well imagined. These experiments were not made with regular patients, but were legitimately conducted, from a feeling that we must know far more about this agent, or abandon its use.

This gas is usually obtained by decomposing nitrate of ammonia by heat. It may be preserved over water, as there will be but little waste after this liquid is once saturated.

Several precautions are to be observed in its preparation. It is much easier to prepare pure ether or chloroform than pure nitrous oxyd. It is sometimes difficult to obtain pure nitrate of ammonia. Here are two specimens, neither fit to be used as ordinarily directed. The gas prepared from this, by the ordinary process, produces a sense of suffocation, and tonic spasm of the muscles of the throat, and sometimes of the respiratory muscles, these symptoms continuing with greater or less severity, in some cases for several days. The salt contains a soluble chloride. The other specimen which I show you does not contain a chloride, but when ordinarily used, yields a gas but little less suffocating than the former. The muscular spasm of the throat is not so continuous as in the former case, but quite as prolonged. Of course the experiments with such agents have been but few. The latter specimen yields pure nitrous oxyd, after about one-fourth of it has been evaporated. (It is less difficult to obtain the pure salt now.—W )

But the use of a pure salt, by no means insures a pure gas. To obtain such a result, several conditions are to be observed. The nitrate is to be decomposed at the proper temperature; and this implies some reliable method of *regulating* the heat. In short, the apparatus should be automatic; for no one can regulate the heat properly on the basis of observation.

The thing to be aimed at is to decompose the nitrate so as to obtain only protoxyd of nitrogen and water, as indicated in this equation :

$$NH^3, NO^5 = 3HO + 2NO.$$

It is difficult, and perhaps impracticable, to obtain exactly this result, as below the proper temperature the order of decomposition is not wholly as indicated by the equation, and of course, in reaching the proper degree of heat, this lower temperature has to be passed. For this reason, the heat should be rapidly raised from the melting point of the salt to the degree of proper decomposition.

By decomposing the nitrate at about 470° Fahrenheit, I have obtained the most satisfactory results; and any temperature between 465° and 480° will afford good gas, if proper care is taken in other respects.

By running the heat too high, a part of the nitrate is decomposed so as to yield binoxyd of nitrogen, sometimes called nitric oxyd, as indicated thus:

$$NH^3, NO^5 = 2NO^2 + HO + H^2.$$

As nitrous oxyd is formed at the same time, it and the free hydrogen form an explosive mixture, and a series of infinitesimal explosions result, agitating the liquid differently from ebullition or effervescence. This condition is readily detected by the practiced eye.

The nitric oxyd, thus formed, is a very poisonous gas, and is very rapidly converted into nitrous acid, which as rapidly passes into nitric acid, by increase of oxydation. This is a much lighter gas than nitrous oxyd, and is far less soluble in water; consequently contrary to the popular opinion and the statements of some writers on the subject, it can not be removed from nitrous oxyd by washing, or passing the mixture through water. A mixture of these two gases becomes more and more unfit for use, by repeated and prolonged washings.

In administering the nitrous oxyd, the patient must not be smothered. This is an important, yet much neglected point, in the use of any anæsthetic. The apparatus ought to be so arranged that respiration is not in the least obstructed. This inhaler is defective. The expiration is considerably retarded, which is a very serious fault. A tube of sufficient diameter, with proper valves, without wings or flanges is the best "inhaler." The patient should be seated in a *very* comfortable position; for with a few inhalations of the gas sensation is so much exalted that trivial inconveniences become painful and very annoying. When the patient has taken the inhaler into his mouth, hold open the valve, and have him make a few full inhalations of air, for the purpose of removing *all* carbonic acid from the air cells. This is practically of very great importance. The first full inspiration of nitrous oxyd seems almost to overwhelm the lungs. The rush of carbonic acid into the air cells is very great. Hence it is nearly always best, after a single inspiration, to open the valve, and let the patient take one or two breaths of air; and through the whole process of administration, whenever, by flushed features or otherwise, there is the least indication of suffocation, admit air freely till relief is afforded. After a little time the rush of carbonic acid abates, and the admission of air is not called for. There should be nothing like forcing the patient to take the gas, such as holding the lips, etc.; for when the gas is pure he *wants to take it.* And when pure and properly administered, it produces neither delirium nor darkening of the complexion. I have sometimes used it regularly for months, without seeing either of these symptoms. Both are caused by the presence of carbonic acid in the air-cells, and not by nitrous oxyd.

Respiration is rendered much slower by the inhalation of nitrous oxyd, being often reduced to six or seven, and even to three or four inspirations to the minute, and this usually without any sense of suffocation or approaching asphyxia. The retarded respiration sometimes continues a considerable time after the operation, causing the patient to feel a sense of prostration; but this is not commonly the case. When a second operation is necessary, it is best to wait till respiration has been re-established.

### FILLING TEETH.

Dr. Taft suggested that perhaps one of the most common faults in practice in filling teeth, is a want of thoroughness in manipulation, too many points are passed over without sufficient attention. How often does the thought occur to all of us, "Oh, well that will do," and especially when we are hurried and fatigued. Failure will often enough ensue, when the highest skill exercises the greatest care. Let all things be done in the most thorough manner possible. I would not intimate that there is but one good way or efficient method of performing this operation in our practice, there will be differences here, as well as everywhere else. I will for a moment consider the operation of filling proximal cavities of the teeth, and will direct attention to but one feature of this, viz.: the separation. This in the molars and bicuspids is usually effected by cutting and filing from the proximal surfaces in which the cavity is situated, till a V shaped space is formed, cutting in this manner till ample space is secured through which to operate, and firm borders of the lateral walls obtained, and then filling the cavity only flush with its borders.

While in some cases this perhaps is the best method, there are others in which we think a different one preferable; for instance, when there are but small or medium sized cavities, the lateral walls thick and firm; it is better to make only separation enough between the teeth to make a good finish upon the proximal surface of the filling; space enough to receive a thin finishing file and tape will be sufficient, and this in the majority of cases, can be obtained by wedging. An entrance into the cavity for the introduction of the filling, should be made by cutting down through the masticating surface of the tooth, into the decayed cavity. This cutting should usually be made as far toward the center of the crown as the decay extends.

By this method the natural form of the tooth is restored, and the ability to masticate is not impaired, and the difficulties arising from a large V shaped space are obviated, and the facility of performing an operation in this manner is equal to, if not greater than other methods.

Dr. Driggs, It is one thing to know how to perform, but quite another to do it. I still adhere to the old method of preparing cavities, have selected the old masters as my copies. There is a disposition in the profession to avoid extremes—to refuse to operate upon teeth that can not be saved with certainty. My practice is to cut down all thin, friable walls or edges, except perhaps upon the incisors; in the molars always cut away the thin edges or walls, and do not make much protrusion of the gold; do not attempt to make contour fillings. I do not believe they will ultimately prove permanent; in a small proximal cavity of a molar, do not think the best method of effecting an entrance into the cavity is by cutting down from the masticating surface of the crown, but obtain an entrance by a separation of the teeth, making as little cutting of the tooth as possible, to secure a good entrance into the cavity. I am in favor of conservative filling; do not extract all badly decayed teeth, nor do I always cut down a large portion of the tooth; but aim to have strong walls, and fill flush with their edges, and in favorable cases, build out somewhat so as to make a convex surface to the filling.

Dr. H. A. Smith, I regard the principles announced by Dr. Driggs, in the main correct; there are, however, various methods of making very good operations.

I desire further information in regard to the new preparations of gold for filling. I am somewhat in doubt as to the advantages claimed for them; and shall be glad to know that they are all right.

Dr. Goddard, I have used about two ounces of "Morgan's Plastic Gold," and chiefly in connection with soft foil; but am not yet fully satisfied with the tests I have made. I fear from some things I have seen, that it may fail; but, as with many other things, so with this, time will decide.

About eighteen months ago, I operated upon a superior central incisor, a large cavity upon its anterior surface; after properly forming the cavity, I fitted into it as neatly as I could a piece of natural tooth; this I set in the cavity with os-artificial, it is yet worn without any apparent change. Can not operations of this kind be frequently made?

Dr. Watt, I have in three instances performed operations in the same manner as described by Dr. Goddard; all were very satisfactory.

Dr. DeCamp, This is a subject of great importance to all, and especially to the younger members of the profession.

Gold is doubtless the best material known for filling teeth; but there may be a diversity of opinion as to the form or condition. I have used it in every form in which it has been presented; foil, crystal, sponge and shred. Some of these, I have observed, discolor after being in use for a time. I have attained better success, made more reliable fillings with soft gold foil, than with anything else. In superficial and difficult cavities, I usually prefer adhesive gold. The form, size, and location of the cavity to be filled, will to some extent determine the kind of gold; I fill extensively with blocks or cylinders.

Dr. Watt, There is a great want of uniformity in all the preparations of gold. This arises from two sources, viz.: the mechanical and chemical manipulation.

Much of our failure to secure good results with new materials and new forms, arises from a want of the proper knowledge to direct their use. Gold perfectly crystalized is, I think, the best form in which it has yet been used. The production of this requires a high degree of chemical knowledge. Crystal gold can not be made as cheaply as foil. Far greater rapidity of execution in filling is attained with crystal gold than with gold foil.

Dr. Arrington, I am not exclusive in my practice, nor in my teaching. I used "Lamb's Gold" for a time, I then thought it good; but have found several samples of it very imperfect, which illustrates what Prof. Watt has said upon that point. I regard "Watt's Sponge Gold" as better than any kindred preparation that I have used; but I have my fears that it sometimes clogs, and does not conform to all inequalities. I use perhaps non-adhesive foil more than any thing else; use nothing for filling but gold and os-artificial. Have put in these fillings in the manner described by Dr. Goddard; I used porcelain, but I now think the natural tooth would be better. I have always condemned the use of amalgam for filling teeth, because it is not reliable, and because of its pernicious influence upon the profession. I do not use "Hill's Stopping," because "os-artificial" is better. In many cases the latter makes excellent fillings, and under no circumstances can it result in injury.

Dr. G. W. Field, By permission, I would ask, if there is any efficient treatment for Dental exostosis, and if so, what it is?

Dr. Watt, This affection is easily treated; it is simply a hypertrophy of the cementum. By this growth pressure is made upon the surrounding parts, these are absorbed, and the growth goes on and oftentimes branches of nerves are impinged upon, and neuralgia occurs. I have found nothing better for treatment of this affection, than iodide of potassium. This agent acts especially upon abnormal growths, breaking down and destroying them. Healthy tissue resists the action of this agent correspondent to the vigor of the vitality, while abnormal tissue is acted upon in almost any case. Iodide of potassium may be taken in from 10 to 30 grain doses three times daily.

There are cases of exostosis, doubtless, in which extraction of the affected tooth or teeth is the only remedy.

Dr. J. Taylor, I have found patients who could not tolerate iodide of potassium.

Dr. Watt, Bromide of potassium may be substituted for the iodide. It may be taken in 20 to 30 grain doses twice daily.

